# Understanding the role of oxidative stress in the incidence of metabolic syndrome and obstructive sleep apnea

**DOI:** 10.1186/s12902-021-00735-4

**Published:** 2021-04-21

**Authors:** Behnam Kargar, Zahra Zamanian, Majid Bagheri Hosseinabadi, Vahid Gharibi, Mohammad Sanyar Moradi, Rosanna Cousins

**Affiliations:** 1 Department of Pharmacology, Medical School, Shiraz University of Medical Sciences, Shiraz, Iran; 2 Department of Occupational Health, School of Health, Shiraz University of Medical Sciences, Shiraz, Iran; 3 School of Public Health, Shahroud University of Medical Sciences, Shahroud, Iran; 4 Department of Psychology, Liverpool Hope University, Liverpool, UK

**Keywords:** MetS, Obesity, OSA, Oxidative stress, Work environment

## Abstract

**Background:**

Understanding the causes and risk factors of metabolic syndrome is important for promoting population health. Oxidative stress has been associated with metabolic syndrome, and also obstructive sleep apnea. These are two diseases which have common prognostic characteristics for heart disease. The aim of this study was to examine the role of oxidative stress in the concurrent presence of metabolic syndrome and obstructive sleep apnea in a working population.

**Methods:**

Participants were 163 artisan bakers in Shahroud, Iran, routinely exposed to significant heat stress and other oxidative stress indicators on a daily basis as part of their work. Using a cross-sectional design, data relevant to determining metabolic syndrome status according to International Diabetes Federation criteria, and the presence of obstructive sleep apnea according to the STOP-Bang score, was collected. Analyses included hierarchical binary logistic regression to yield predictors of the two diseases.

**Results:**

Hierarchical binary logistic regression showed that oxidative stress – alongside obesity, no regular exercise, and smoking – was an independent predictor of metabolic syndrome, but not obstructive sleep apnea. Participants who were obese were 28 times more likely to have metabolic syndrome (OR 28.59, 95% CI 4.91–63.02) and 44 times more likely to have obstructive sleep apnea (OR 44.48, 95% CI 4.91–403.28). Participants meeting metabolic syndrome criteria had significantly higher levels of malondialdehyde (*p* <  0.05) than those who did not. No difference in oxidative stress index levels were found according to obstructive sleep apnea status.

**Conclusions:**

Our findings suggest that oxidative stress contributes to the onset of metabolic syndrome, and that obstructive sleep apnea is involved in oxidative stress. Whilst obesity, exercise, and smoking remain important targets for reducing the incidence of metabolic syndrome and obstructive sleep apnea, policies to control risks of prolonged exposure to oxidative stress are also relevant in occupations where such environmental conditions exist.

**Supplementary Information:**

The online version contains supplementary material available at 10.1186/s12902-021-00735-4.

## Background

Obstructive sleep apnea (OSA (is a sleep-related breathing disorder. It is caused by repetitive obstruction of the upper respiratory tract during sleep which can lead to decreased sleep quality, hypoxia, hypercapnia, increased negative pressure inside the chest cavity, and increased sympathetic activity. Breathing stoppages, snoring, daytime sleepiness, and early morning headaches are symptoms of this disorder [1]. Epidemiological studies have shown that patients with OSA are more likely to develop cardiovascular disease, hypertension, hyperlipidemia, insulin resistance and atherosclerosis [2]. The repetitive cycle of upper airway closure and opening results in a frequent hypoxia / reoxygenation process can induce oxidative stress by an overproduction of free radicals [3]. Oxidative stress and OSA may share some common pathophysiological characteristics such as atherosclerosis, cardiovascular disease, and cerebral complications [4].

There are also studies suggesting that oxidative stress plays a key role in the metabolic pathogenesis of metabolic syndrome (MetS) [5]. The global epidemic of MetS, closely linked to symptoms such as obesity, insulin resistance, high blood pressure, impaired glucose tolerance or diabetes, hyperinsulinemia and dyslipidemia, has significant negative socioeconomic impacts [6]. The presence of three of these symptoms is required for a diagnosis of MetS [7]. Understanding the underlying causes and risk factors of MetS can play a significance role in lifestyle, planning and resource allocation. This is important as most researchers believe that MetS can be considered as a prognostic sign of cardiovascular disease as well as type 2 diabetes [8].

Normal metabolism includes the continuous production of reactive oxygen species (ROS) and free radicals. Redox equilibrium is vital for maintaining health. Oxidative stress is seen when there is an imbalance in redox characteristics leads to the overproduction of reactive species or a reduction in the capability of endogenous antioxidant systems. Both are damaging to metabolic processes and consequently to health [9]. Reactive oxygen species (ROS) levels are increased in obesity. This is the major component of MetS which can be decreased by weight loss [10]. In addition, there is evidence that ROS may contribute to the development of insulin resistance, which is an important precursor of MetS [8]. Similarly, the review paper by Ando et al. [11] suggested that ROS may also play a role in the pathogenesis of high salt intake-induced hypertension and cardiovascular diseases, making ROS one of the key factors in the development of metabolic syndrome.

Hopps et al. [12] introduced assessment of oxidative status in people with MetS as a solution to determine the complexity of risk factors for cardiovascular diseases. According to the review article by Roberts et al. [5], oxidative stress is associated with many components of metabolic syndrome, and it is the causes of metabolic syndrome risk factors such as insulin resistance, high blood pressure, increased lipid levels, inflammation and endothelial dysfunction. Numerous studies have shown that regular physical activity and dietary modifications can prevent oxidative stress, nevertheless the molecular mechanism by which physical activities and dietary modifications prevent oxidative stress in metabolic syndrome is still unknown [5].

The rationale for considering MetS and OSA together followed from reports of an association of OSA and metabolic disorders. For example, Watz et al. [13] showed that the risk of metabolic syndrome was 9 times higher in people with OSA, and the treatment of OSA plays an important role in the management of MetS [14]. To date, no study has considered the role of oxidative stress in concurrent presence of MetS and OSA, and the relationship of oxidative stress with these two disorders.

Bread making is an occupation which presents many health hazards, particularly for bakers in Iran who use traditional artisan methods. Hazards include chronic exposure to high levels of heat from open ovens, high levels of humidity, flour dust, shift working, and significant manual labor: all factors that can affect oxidative stress indicators. Of note Bolghanabadi et al. [15] indicated high heat stress in more than 80% of bakers working in Neyshabour. Similarly, a study in Ahvaz [16], found most of the bakers who acted as participants to be a risk of heat stress. Both studies made these assessments based on wet-bulb globe temperature and made reference to the heat stress standard ISO 7243, which has global application [17]. Therefore, the aim of this study was to investigate the role of oxidative stress in the incidence of MetS and OSA using a sample of traditional artisan bakery workers towards understanding the health risks.

## Methods

### Design, setting and participants

The target population of this cross-sectional study worked in bakeries in Shahroud, Iran. The city had 95 active bakeries with 270 workers. Permission to recruit on the basis on informed consent was provided by 74 bakery units. One hundred sixty-three male workers in these bakeries who met the criteria for the study and provided written consent entered the study. Inclusion criteria were having at least 12 months of work experience and working full-time. Exclusion criteria were illnesses such as hypertension, kidney disease and heart failure, the use of antioxidant medication or supplements, and not having routine exposure to bakery ovens.

### Measures and procedure

Demographic information relating to the age, marital status, level of education, smoking, and work experience of each participant was collected in person using a standard questionnaire. (The questionnaire is available as a Supplementary File). Each participants’ weight and height were measured using precision instruments. Body Mass Index (BMI) was calculated (weight in kg / height in m^2^) and classified according to the World Health Organization nutritional status: *Underweight*, if BMI < 18.5, *Normal* if BMI 18.5–29.9, *Obese* if BMI ≥ 30.0. Waist circumference was measured with a non-elastic tape (measuring accuracy ±1 mm) without imposing any pressure on the body at the end of normal exhalation. Participants were asked to abstain from caffeine for 30 min prior to blood pressure measurement. Blood pressure was measured twice by trained individuals using a 15-min interval. The blood pressure recorded was the average of the two readings.

### Obstructive sleep apnea

The STOP-Bang questionnaire was used to identify participants with symptoms of obstructive sleep apnea [18]. This screening tool has been translated into Persian and appropriate validity and reliability for OSA confirmed [19]. STOP-Bang comprises eight yes / no questions related to age ≥ 50 years, sex, snoring loudly, feeling sleepy during the daytime, been observed to stop breathing during sleep, high blood pressure, BMI ≥ 35 kg/m^2^, and neck circumference ≥ 40 cm. Endorsement of three of the eight items is indicates a high risk of OSA.

### Metabolic syndrome

The International Diabetes Federation criteria [7] were used to assess MetS (see Table 1).

**Table 1:** International Diabetes Federation criteria [7] for metabolic syndrome

For someone to be classified as having MetS they must have: Central obesity (waist circumference for this sample ≥ 94 cm), and at least two of the four components below	
**Hyperglycemia**	Fasting glucose ≥100 mg/dL or previously diagnosed Type 2 diabetes
**Plasma triglycerides**	TG ≥ 150 mg/dL or existing treatment for this lipid abnormality
**HDL cholesterol**	< 40 mg/dl or existing treatment for this lipid abnormality
**Hypertension**	≥ 130 mmHg systolic or ≥ 85 mmHg diastolic or treatment of previously diagnosed high blood pressure

Blood samples were collected after a 12-h fasting period. Blood sugar levels were measured using a fasting plasma glucose test (enzymatic methods: Helena Biosciences). Plasma triglycerides, high-density lipoproteins (HDL), and cholesterol levels were derived from enzymatic end-point measurements performed using enzyme reagent kits and a Swiss-made automated analyzer (Lysis). The quality of the devices was checked before and after the tests. The repeatability of tests was confirmed (*r* = 0.995).

### Oxidative stress indices

Three biochemical markers of oxidative stress were measured in serum samples. Malondialdehyde (MDA) is one of the major end products of the oxidation of unsaturated fatty acids by free radicals. It is formed by a group of free radicals called hydroxyl radical inducing lipid peroxidation. Raised levels of serum MDA are indicative of oxidative stress [20]. Nitric oxide (NO) is a major contributor to normal endothelial and vascular function. Raised NO levels indicate inflammation, and together with other ROS, NO contributes to oxidative stress [21]. Total antioxidant capacity (TAC) assays predominantly measure chain-breaking antioxidant activity. TAC is lowered in oxidative stress states [22]. To assess MDA, NO and TAC, blood samples (5 ml) were centrifuged to separate the serum. These were stored at − 20 °C until laboratory analysis. Oxidative stress indices in the serum samples were assayed using kits (Hangzhou East Biopharm) via a double antibody sandwich enzyme-linked immunosorbent assay (DAS-ELISA).

### Statistical analyses

SPSS statistics version 24 was used for all statistical analyses. Alpha levels were set at 0.05 for T-Tests and ANOVAs, and a significance level of 0.1 was used for the hierarchical binary logistics regressions. Logistic regressions were used to determine the predictive variables of MetS and OSA.

## Results

Mean age of the 163 participants was 45.4 years, 108 (66.3%) were regularly exercising and 141 (87.7%) were non-smokers. 22 (13.50%) participants were obese (BMI ≥ 30 kg/m^2^; mean 33.5 ± 2.2). According to International Diabetes Federation criteria, 20 (12.3%) participants had MetS. According to STOP-Bang criteria, 22 (13.5%) participants had OSA.

Tables 2 and 3 show mean serum levels of MDA, NO and TAC according to MetS status and OSA status respectively. Participants with MetS had significantly higher levels of MDA compared with participants without MetS. There was no difference in the oxidative stress index levels for other comparisons.

**Table 2:** Oxidative stress index levels based on MetS status

MetS	Frequency (%)	MDA (nmol Ml^**−1**^)	NO (L^**−1**^)	TAC (μmol/ L^**− 1**^)
**No**	143 (88)	22.21 ± 13.52	71.78 ± 46.71	2.23 ± 1.97
**Yes**	20 (12)	29.33 ± 19.72	62.49 ± 27.99	2.39 ± 2.85
***p*** **value**	–	**< 0.05**	NS	NS

**Table 3:** Oxidative stress index levels based on OSA status

OSA	Frequency (%)	MDA (nmol Ml^**−1**^)	NO (L^**−1**^)	TAC (μmol/ L^**− 1**^)
**No**	141 (87)	28.76 ± 19.19	71.72 ± 46.91	2.24 ± 1.97
**Yes**	22 (13)	26.54 ± 19.41	63.82 ± 28.52	2.31 ± 2.76
***p*** **value**	–	NS	NS	NS

Tables 4 and 5 shows data analyses using the backward log-likelihood ratio method of hierarchical binary logistic regression. These analyses confirmed a significant difference between some demographic variables and the main effects of the study variables including oxidative stress parameters.

**Table 4:** MetS predictors based on logistic regression

Variable	Classification	B	Odds Ratio (OR) (95% CI)	***p-***value
**BMI (kg/m**^**2**^**)**	Normal	*ref*	*ref*	*ref*
	Underweight	1.755	1.081 (0.65–1.73)	NS
	Obese	3.353	28.59 (4.91–63.02)	< 0.01
**Regular exercise**	Yes vs No	5.183	1.08 (1.06–1.211)	< 0.01
**Smoker**	No vs Yes	5.52	24.59 (12.26–61.76)	< 0.001
**MDA (nmol Ml**^**−1**^**)**		0.112	1.89 (1.08–4.68)	< 0.05

**Table 5:** OSA predictors based on logistic regression

Variable	Classification	B	Odds Ratio (OR) (95% CI)	***p-***value
**BMI (kg/m**^**2**^**)**	Normal	*ref*	*ref*	*ref*
	Underweight	−16.33	0.81 (0.64–2.42)	NS
	Obese	3.79	44.48 (4.91–403.28)	< 0.001
**Regular exercise**	Yes vs No	2.24	1.106 (1.02–3.48)	< 0.01
**Smoker**	No vs Yes	2.25	9.51 (2.11–42.97)	< 0.01
**Daily work**	≤8 vs > 8 h	2.05	7.42 (1.31–41.95)	< 0.05

To accurately examine the parameters affecting the development of MetS, the role of all the variables was investigated using the logistics regression model (see Table 4). In the second stage, oxidative stress factors were entered in addition to significant demographic variables (< 0.1) in the univariate analysis of the first stage. BMI, regular exercise, smoking and MDA were identified as independent variables and strong predictors in the final model.

The correlation between obesity (OR 28.59, 95% CI 4.91–63.02) and the development of MetS remained significant after regression analysis (*p* <  0.01). In other words, obese bakers were 28 times more likely to have MetS (*p* <  0.01) compared to those of normal weight. Regular exercise also remained significant in the last stage, indicating that people who were not exercising regularly were 8% more likely to develop MetS compared with those who did exercise regularly (OR 1.08, 95% CI 1.06–1.21; *p* <  0.001). We also found that smokers were 24.59 times more likely to develop MetS than non-smokers (OR 24.59, 95% CI 12.26–61.7; *p* <  0.001). MDA remained significant after regression analysis so that an increase of 1 nmol/mL in serum MDA levels increased the risk of developing MetS by 89% (OR 1.89, 95% CI 1.08–4.68; *p* <  0.05).

To accurately examine the parameters affecting the development of OSA, all variables were similarly examined using a logistics regression model (see Table 5). In the second stage, oxidative stress factors were entered into the model in addition to demographic variables with a significance level of 0.1 in the first stage. BMI, regular exercise, smoking and daily work hours were identified as independent variables and strong predictors in the final model.

The correlation between obesity (OR 44.48, 95% CI 4.91–403.28) and the development of OSA remained significant after regression analysis (*p* < 0.01). In other words, obese bakers were 44 times more likely to have OSA compared with those of normal weight. Also, participants who did not exercise regularly were 8% more likely to develop OSA compared with those who did exercise regularly (OR 1.106, 95% CI 1.02–3.48; *p* < 0.01). Smokers were 9.51 times more likely to have OSA than non-smokers (OR 9.51, 95% CI 2.11–42.97;*p* < 0.01). Daily work hours also remained significant after regression analysis in the final stage. Bakers working more than 8 h a day were 7.42 times more likely to develop OSA compared with those whose daily working period was 8 h or less (OR 7.42, 95% CI 1.31–41.95; *p* < 0.05).

## Discussion

The aim of this study was to investigate the role of oxidative stress in the incidence of MetS and OSA to understand the associated health risks. We used a sample of male bakery workers, on the basis that this was an active occupation where exposure to an environment conducive to oxidative stress would permit a suitable investigation. Prevalence rates of MetS and OSA among the 163 male bakers were relatively low, which was in keeping with a sample with relatively low obesity and smoking levels. Of relevance, the rate of MetS in this study (12.3%) was in keeping with the 10.88% reported for the same city in a recent cohort study [23]. Similarly, the prevalence of OSA was close to the recent estimate that one in seven adults in the general population have OSA [24].

We found BMI, regular exercise, smoking, daily work hours and oxidative stress (as measured by malondialdehyde levels) had statistically significant roles in the development of these diseases. However, BMI, regular exercise, and smoking are common prognostic tools in both diseases. This study replicated such findings and for the first time looked at MetS and OSA concurrently to examine the contribution of oxidative stress to understanding these diseases. Hierarchical binary logistics regression analyses were used to allow identification of predictor variables from our cross-sectional data [25]. We found obesity was the biggest predictor of both MetS and OSA. The results from our study also suggest that oxidative stress can *activate* pathways that play an important role in the development of MetS. That is, oxidative stress can be considered an initiator of the MetS process due to the reactions it creates in the body. However, there was no significant association of oxidative stress and OSA.

Our findings that oxidative stress contributes to MetS in obese men is not at odds with the literature. Specifically, adipose tissue can secrete substances that play a major role in triggering MetS inflammation [26]. Macrophage infiltration of adipose tissue, especially visceral fat, is one of the characteristics of mild inflammation in obesity and there is a positive correlation between BMI and the amount of macrophage infiltration within adipose tissue. Macrophages that are activated in adipose tissue can secrete large amounts of pre-inflammatory adipokines [26]. Increased activation of NF-kβv pathways through oxidative stress and adipokine production can contribute to increased adipokine release [27]. Moreover, adiponectin is an important adipokine that plays a major role in the regulation of muscle metabolism as a plasma protein. The beneficial effects of adiponectin include its anti-inflammatory and insulin sensitivity effects [28]. Plasma levels of adiponectin, strongly associated with MetS, are lower in obese people than in non-obese people [29], and people with the lowest adiponectin levels probably have MetS [30]. Leptin is also an important adipokine and is increased in obese people [31]. Leptin can increase oxidative stress and pre-inflammatory factors [27].

The lack of an independent role for oxidative stress in participants with symptoms of OSA was unexpected. There are several potential reasons for this result. First, we have to acknowledge that whilst STOP-Bang is able to appropriately detect OSA [18, 19, 32], it is a screen for OSA. Polysomnography is required to determine severity of OSA. In their cross-sectional study of the role of oxidative stress in OSA, Yamauchi et al. [33] recruited from a sleep disorders clinic and found it was only the group with severe OSA that had high oxidative stress markers. In our sample of artisan bakers, the prevalence of OSA was modest, and we cautiously suggest that the findings may align with those of their mild OSA sample, whilst recognizing the different methodology. Nevertheless, there are other studies that have also shown no association between the incidence of OSA and increased oxidative stress [34, 35]. A potential reason for this evolves from the severity angle, and the thesis of Lavie [3]. Drawing upon a range of studies she suggested that the repetitive cycle of upper airway collapse and recovery results in a frequent hypoxia / reoxygenation process which can *induce* oxidative stress by an overproduction of free radicals. Moreover, support for this involvement comes from evidence that oxidative stress is reduced in OSA patients following continuous positive airway treatment [3]. In sum, we can understand our results do not deny the relationship of MetS and OSA, if we recognize that oxidative stress is involved in each disease, if in a different way. That is, increased severity of OSA increases oxidative stress, and oxidative stress is involved in the pathogenesis of MetS.

To address the challenge of accurate measurement of oxidative stress [22] we used three measures of oxidative stress. These were malondialdehyde (MDA), nitric oxide (NO), and total antioxidant capacity (TAC). We found MDA levels were significantly raised in participants who had MetS compared to those who did not have MetS. There was no difference in the same participants according to their OSA status. MDA is a commonly used biomarker, and whilst this measure of lipid oxidation has been criticized for being prone to artifacts, it has also been considered to have clinical relevance in inflammatory diseases [9]. We did not, however, find an excess of NO, a free radical and a marker of oxidative stress, according to either MetS or OSA. It remains that the measurement of NO is fraught with difficulty because of its low concentrations in serum, and short half-life [36], nevertheless the ELISA assay kits we used were an established methodology.

We also took TAC measures, following the rationale that low levels of TAC suggests oxidative stress, and because it is routinely used in oxidative stress research. We found no difference in TAC according to MetS or OSA status. We expected TAC levels to be decreased relative to the significant rise in MDA levels seen in those participants with MetS, however this was not so. With respect to OSA, although not significant for either biomarker, a negative relationship of MDA and TAC was observed. This is a challenge to explain, as the measures are from the same participants, and warrants further investigation. That said, Young [22] argues that despite its attraction, TAC is not a good measure of oxidative stress because TAC assays actually measure chain breaking antioxidant activity. In summary from this short discussion, we report that despite the challenges of measuring oxidative stress, we are confident the finding of role for MDA in the prediction of MetS is useful. It remains to say, that this finding should be replicated in due course.

As expected, we found that the absence of regular exercise is associated with both MetS and OSA. Regular exercise is known to improve insulin sensitivity and reduce the risk of MetS and obesity-related diseases [37]. There is also evidence that antioxidant enzyme activity is increased in various tissues after aerobic and anaerobic exercise [38]. A regular exercise-induced adaptation can be justified by Hormesis theory [39], and evidence of a decrease in the production of free radicals after 12 weeks of exercise [40]. Smoking was another prognostic tool in both diseases. Some studies have shown that smoking can contribute to increased abdominal obesity [41] which could be the link.

### Strengths and limitations

Strengths of this study are a substantial sample of relatively healthy bakers doing very similar work in conditions which allowed us to consider the new variables we were interested in – oxidative stress indicators – in relation to our research questions. We were able to show the role of MDA in the prediction of MetS despite the prevalence of MetS in this sample being relatively low. Similarly, we were able to suggest that oxidative stress does not act as a precursor to OSA, but that OSA is likely to increase oxidative stress.

An important limitation for our study was the use of a screening tool for identification of OSA, rather than the gold standard methodology of polysomnography. Nevertheless, this half survey (STOP), half personal physiologic measures (Bang) screen was developed and validated to support quick and inexpensive assessment of this disease [18], as discussed above. It has been used extensively in research. Moreover, a meta-analysis of 17 studies including 9206 people who completed the STOP-BANG questionnaire then underwent polysomnography concluded STOP-BANG has excellent positive and negative predictive ability to identify OSA [32]. Another limitation was the use of a male only population. This was a consequence of the study design. We recognize the need for similar studies to replicate our findings. Such studies could be extended to include females.

## Conclusions

The present study indicates that oxidative stress is implicated in the development of MetS in bakery workers. No significant association was found between oxidative stress and the incidence of OSA. Obesity, smoking and exercise remain important targets for reducing the risk of metabolic syndrome and obstructive sleep apnea and maintaining good health. Policies to control risks of prolonged exposure to oxidative stress factors such as heat, humidity, flour dust, shift work and significant manual labor are important to ameliorating the risk of metabolic syndrome in bakers and other occupations with these environmental conditions.

## Supplementary Information

**Additional file 1.**

## Data Availability

The datasets used and analyzed during the current study are available from either of the corresponding authors on reasonable request.

## Abbreviations

BMI: Body mass index
CI: Confidence interval
HDL: High-density lipoproteins
MDA: Malondialdehyde
MetS: Metabolic syndrome
NO: Nitric oxide
OR: Odds ratio
OSA: Obstructive sleep apnea
ROS: Reactive oxygen species
TAC: Total antioxidant capacity
HDL: High-density lipoprotein
