# Supplementary material for: Understanding the role of oxidative stress in the incidence of metabolic syndrome and obstructive sleep apnea
Source: BMC Endocr Disord. 2021 Apr 21;21:77. doi: 10.1186/s12902-021-00735-4 (PMC8059172; doi:10.1186/s12902-021-00735-4)
Supplement: Supplementary file 1 — Additional file 1. [file 12902_2021_735_MOESM1_ESM.docx]

| **SOCIODEMOGRAPHIC QUESTIONNAIRE**  Study among Artisan Bakers in Shahroud |
| --- |
| NOTE:   - Participation in this study is completely optional. - Your information will be kept completely confidential by the researchers. - Please answer all the questions as accurately as possible. |

**First name:………………. Last name:…………. Identification Code:………**

**Type of bakery:………….. Address:**

**Phone number:**

1. What is your gender?

- Male
- Female
- Other

1. Do you currently use tobacco?

- Yes, on a regular basis
- Yes, but only once a week
- No, I quit
- Never

1. Do you have any of the following health issues?

- Obesity
- Heart disease
- Asthma
- Cancer
- Type 1 diabetes
- Type 2 diabetes
- Autoimmune disease
- Hypertension
- Kidney disease
- Other (please specify) ……………………

1. What is your marital status?

- Single
- Married
- Other

1. Do you exercise regularly? (i.e. You have exercised for at least 30 mins, raising a light sweat, twice a week for over a year)

- Yes
- No

1. What is highest educational qualification? …………..
2. What is your date of birth? (Date, Month, Year) ………………
3. How many hours do you work each week? …….………..
4. How many years of bakery work experience do you have? ……….……

| **STOP-BANG Sleep Apnea QUESTIONNAIRE**  Study among Artisan Bakers in Shahroud |
| --- |

| **Based on the researcher's measurements according to the protocol** | |
| --- | --- |
| **Weight (kg):** | **Height (m):** |
| **Neck circumference (cm):** | |
| **BMI (kg/m^2^):** | |

| **STOP** | | |
| --- | --- | --- |
| Do you **SNORE** loudly (louder than talking or loud  enough to be heard through closed doors)? | Yes | No |
| Do you often feel **TIRED**, fatigued, or sleepy during  daytime? | Yes | No |
| Has anyone **OBSERVED** you stop breathing during  your sleep? | Yes | No |
| Do you have or are you being treated for high blood  **PRESSURE**? | Yes | No |

| **BANG** | | |
| --- | --- | --- |
| **BMI** more than 35kg/m^2^? | Yes | No |
| **AGE** over 50 years old? | Yes | No |
| **NECK** circumference > 16 inches (40cm)? | Yes | No |
| **GENDER**: Male? | Yes | No |

| **Record laboratory results**  Study among Artisan Bakers in Shahroud |
| --- |

| **Result** | **Variable** | | **NO.** |
| --- | --- | --- | --- |
|  | Fasting glucose (mg/dL) | | 1 |
|  | TG :Plasma triglycerides (mg/dL) | | 2 |
|  | HDL cholesterol (mg/dL) | | 3 |
|  | systolic | Hypertension (mmHg) | 4 |
|  | diastolic |  | 5 |
| **Researchers ask participants the following two questions:** | | | |
|  | 1. Are you receiving treatment for a specific disease? (Please specify) | | |
|  | 1. Are you taking any medication? (Please specify) | | |
|  | MDA (nmol Ml^-1^) | | 6 |
|  | NO (L^-1^) | | 7 |
|  | TAC (μmol/ L^-1^) | | 8 |
